# Variation sites at the *HLA-G* 3’ untranslated region confer differential susceptibility to HIV/HPV co-infection and aneuploidy in cervical cell

**DOI:** 10.1371/journal.pone.0204679

**Published:** 2018-10-02

**Authors:** Fernanda Silva Medeiros, Albert Eduardo Silva Martins, Renan Garcia Gomes, Sávio Augusto Vieira de Oliveira, Stefan Welkovic, Magda Maruza, Maria Luiza Bezerra Menezes, Ricardo Arraes de Alencar Ximenes, George Tadeu Nunes Diniz, Eduardo Antônio Donadi, Norma Lucena-Silva

**Affiliations:** 1 Department of Immunology, Laboratory of Immunogenetics, Aggeu Magalhães Institute, Oswaldo Cruz Foundation, Recife, Brazil; 2 Integrated Health Centre Amaury de Medeiros (CISAM), University of Pernambuco, Recife, Brazil; 3 Hospital Correia Picanço, Health Secretariat of Pernambuco, Recife, Brazil; 4 Maternal and Child Department of the Faculty of Medical Sciences—University of Pernambuco (UPE), Recife, Brazil; 5 Department of Tropical Medicine, Federal University of Pernambuco (UFPE), Recife, Brazil; 6 Department of Public Health, Laboratory Computational Methods, Aggeu Magalhães Institute, Recife, Brazil; 7 Department of Medicine, School of Medicine of Ribeirão Preto, University of São Paulo (USP), Ribeirão Preto, Brazil; 8 Pediatric Oncology Service, IMIP Hospital, Recife, Brazil; Istituto Nazionale Tumori IRCCS Fondazione Pascale, ITALY

## Abstract

Post-transcriptional regulatory elements associated with transcript degradation or transcript instability have been described at the 3’ untranslated region (3’UTR) of the *HLA-G* gene. Considering that HPV infection and aneuploidy, which causes gene instability, are associated with cervical cell malignancy, as well as the fact that HIV infection and HLA-G may modulate the immune response, the present study aimed to compare the frequencies of *HLA-G* 3′UTR polymorphic sites (14-base pair *insertion/deletion*, +*3142C/G*, and +*3187A/G*) between 226 HIV^+^ women co-infected (n = 82) or not with HPV (n = 144) and 138 healthy women. We also evaluated the relationship between those *HLA-G* 3’UTR variants and aneuploidy in cervical cells. HPV types and HLA-G polymorphisms were determined by PCR and sequencing of cervical samples DNA. Aneuploidy in cervical cell was measured by flow cytometry. The *HLA-G* 3’UTR 14-bp *ins/del* was not associated with either HIV nor HIV/HPV co-infection. The *+3142G* allele (*p* = 0.049) and *+3142GG* genotype (*p* = 0.047) were overrepresented in all HIV-infected women. On the other hand, the *+3187G* allele (*p* = 0.028) and the *+3187GG* genotype (*p* = 0.026) predominated among healthy women. The +*3142G* (*p* = 0.023) and *+3187A* (*p* = 0.003) alleles were associated with predisposition to HIV infection, irrespective of the presence or not of HIV/HPV co-infection. The diplotype formed by the combination of the +3*142CX* (*CC* or *CG*) and +3*187AA* genotype conferred the highest risk for aneuploidy in cervical cell induced by HPV. The *HLA-G* 3’UTR +*3142* and +*3187* variants conferred distinct susceptibility to HIV infection and aneuploidy.

## Introduction

The human leukocyte antigen-G (HLA-G) is a non-classical major histocompatibility complex molecule, characterized by low protein variability and restricted tissue distribution. HLA-G was initially identified in cytotrophoblast cells and plays an important role in the modulation of the immune response by inhibiting maternal NK and CD8^+^ T cytotoxic cells, and inducing IL-10 producing T regulatory cells at the maternal-fetal interface, which are crucial events for fetus survival [1,2]. Low level of placental HLA-G has been associated with the development of eclampsia and repeated abortion episodes [3]. The immune checkpoint HLA-G molecule has a well-recognized role on the inhibition of several functions of the innate and adaptive immune responses [4].

HLA-G expression may be differentially modulated by variation sites especially those at the regulatory regions of the *HLA-G* gene (promoter and 3’ untranslated sequence) [5–7]. Three variation sites at the *HLA-G* 3’ untranslated region (3’UTR) are important for the post-transcriptional regulation of *HLA-G*: the 14-base pair (14-bp) *insertion/deletion* variants influence *HLA-G* mRNA stability [8,9], the *+3142C/G* variants regulate *HLA-G* expression mediated by specific microRNAs [10,11], and the *+3187A/G* variants affect *HLA-G* mRNA degradation [12]. Polymorphic sites observed at *HLA-G* 3’UTR have also been associated with risk to develop cancer [13], autoimmunity disorders [14,15], and infectious diseases [11,16], among other conditions.

*HLA-G* 3’UTR alleles have been associated with susceptibility to human immunodeficiency virus (HIV) infection in adults [6,17] and in perinatal HIV transmission [18]. HIV^+^ women have a higher risk of developing human papillomavirus (HPV) co-infection, which is the major cause of human cervical cancer [19,20]. HPV infection [9,11,21–23] and HPV-associated cervical cancer [24–26] have also been associated with the presence of polymorphic sites at the *HLA-G* gene, irrespective of HIV infection.

In a previous study, we reported a high frequency (47.5%) of HPV infection among HIV^+^ women from the state of Pernambuco, northeast Brazil, and 59% of those women harbored high-risk HPV types. Despite the high frequency of HPV infection, we observed a low occurrence of cervical lesions in the HIV^+^ women, which was attributed to the high adherence to antiretroviral therapy [27]. In addition, we observed an association between cellular intraepithelial neoplasia and the presence of aneuploid cells, which causes genomic instability in cervical cells of HIV^+^/HPV^+^ women [28].

Considering that, i) the HPV infection is associated with several stages of cervical cell differentiation, ii) the HIV infection and HLA-G may modulate the immune response, iii) the *HLA-G* 3’UTR segment has several post-transcriptional control elements for HLA-G expression, and iv) aneuploidy is one of the first events in cervical cancer development, the present study aimed to compare the frequencies of *HLA-G* 3′UTR polymorphic sites (*14-bp ins/del*, +*3142C/G* and +*3187A/G*) between HIV^+^ women co-infected or not with HPV and healthy women, and to evaluate the association of these *HLA-G* 3’UTR variants with aneuploidy in cervical cells.

## Methodology

### Study design and ethical consideration

We performed a case-control study comprising 226 HIV^+^ women followed-up at the Correia Picanço and the Integrated Health Centre Amaury de Medeiros (CISAM) Hospitals of Recife, capital of the state of Pernambuco, northeast Brazil. Of the 226 HIV^+^ patients, 82 (36.3%) were co-infected with HPV and 144 (63.7%) were not co-infected with HPV. In parallel, we studied 138 HIV^-^ healthy women followed-up at CISAM for routine examination. The study was approved by the Research Ethics Committee of CISAM under the protocol CAEE: 0011.0.250.000–05. All women agreed to participate in the study and signed a free and informed consent before any assessment.

### Data collection

All women answered a questionnaire for clinical and epidemiological assessment, before undergoing colposcopy and cytological examination. Colposcopy was performed according to the criteria of the International Nomenclature of Colposcopy Aspects of Rome [27–28]. Endocervical samples collected using cytobrush were used for cytological analysis, detection of HPV co-infection and immunogenetic studies. Cervical biopsy was performed whenever colposcopic alterations were present and the cervical tissue fragments were preserved in 10% formalin for histopathological analysis. The clinical procedures and the analysis and interpretation of the cervical cancer screening followed the recommendations of the Brazilian guidelines for cervical cancer screening [29]. Details on clinical characterization of HIV^+^ infected patients have been previously described [27]. Healthy women were not tested for HPV.

### Detection of HIV/HPV co-infection

Genomic DNA was extracted from cervical samples using proteinase K (Invitrogen, CA, USA) following the protocol described by Martins et al. 2014 [27]. Subsequently, the DNA samples were amplified by polymerase chain reaction (PCR), using the human glyceraldehyde phosphate dehydrogenase (*GAPDH*) gene to evaluate sample quality. *GAPDH*-positive samples were then subjected to another PCR using the degenerate primers, MY09 and MY11, complementary to the *L1* region of the HPV genome [30]. PCR products were visualized on a 2% agarose gel stained with 0.5 μg/mL ethidium bromide. The presence of a 450 bp DNA fragment confirmed HIV/HPV co-infection. HPV typing was determined by sequencing the HPV-amplified DNA using the Big Dye Terminator kit and the ABI3100 Genetic Analyzer (Applied Biosystems, Foster City, CA). Sequencing chromatograms were visualized using the MEGA 5.0 software [31] for evaluation of the sequence quality. Analysis of sequence data and sequence similarity searches were performed using the Blast-N tool of the National Center for Biotechnology Information (NCBI) (http://blast.ncbi.nlm.nih.gov/Blast.cgi).

### Aneuploidy in cervical cell

Cervical cells were lysed using Pharm Lyse lysing buffer (Becton Dickinson, New Jersey, USA) for 30 minutes prior incubation with RNA solution (100 μg/mL) and propidium iodide. DNA fluorescence was measured by flow cytometry (laser excitation at 488 nm and emission above 600 nm), and DNA index was estimated through by comparison the ratio of the DNA content of cells analyzed with labeled blood diploid cells using the ModFitLT V3.0 software (Verity Software House Inc., Topsham, USA). The presence of two peaks on a histogram with a DNA index greater than 1.16 (hyperploidy) or less than 1.00 (hypoploidy), each with more than 10% of the cell population analyzed in the area corresponding to G0–G1 of the cell cycle in the sample, was considered to be aneuploidy [28].

### Polymorphism of the *HLA-G* 3’ untranslated region

Polymorphic sites between the *+2945* and *+3259* positions of the *HLA-G* gene were identified as described by Castelli et al. (2010) [32]. Briefly, amplification was performed in a final volume of 25 μL containing 25 pmol of each primer (HG08F and HG08R), 50 ng of genomic DNA, 1x PCR buffer (20 mM Tris-HCl, 50 mM KCl, 1.5 mM MgCl_2_), 0.3 mM dNTP, and 1 U *Taq* DNA polymerase (BioTools, Madrid, Spain). The cycling conditions included one cycle at 94°C for 3 min, followed by 40 cycles at 94°C for 30 s, 60°C for 60 s, 72°C for 30 s, and a final extension step at 72°C for 7 min. A 350 bp amplified fragment was sequenced with the reverse primer, HG08R. All polymorphic sites were identified using SeqMan software version 7.0.0 (DNAStar Inc., Madison, USA) and individually annotated.

### Data analyses

The clinical-epidemiological and laboratory data were analyzed using the Excel 2007 software. HPV types were classified according to their phylogenetic characteristics, as previously reported by de Villiers et al. (2004) [33]. Thirty (43.0%) viral isolates were classified as low-risk (HPV 6, 11, 61, 54, 62, 71, 72, 81, 84, 85, 86) and forty (57.0%) were classified as high-risk (HPV 16, 18, 31, 33, 45, 52, 53, 56, 58, 59, 66, 69, 70, 82). We were able to present the frequency of virus types in only 70 patients. The lack of HPV genotype information for 14.6% of the cases was due to the low quality of the sequencing chromatogram, including cases of mixed-HPV subtype infection [27]. However, for the study of the frequencies of *HLA-G* 3'UTR variants, we were able to evaluate 82 HIV^+^/HPV^+^ patients (not all HPV-typed). Data were analyzed using the statistical software Stata 10.0 (Stata Corp LP, USA). Odds ratios (OR) and their respective 95% confidence intervals (CI95%) and *p*-values (χ^2^ test and the likelihood ratio) were estimated in the univariate analysis. Associations with *p* < 0.05 were considered significant. Variables with *p* < 0.25 were included in the multivariate analysis. The non-parametric Mann-Whitney *U* test was used when appropriate. The allele and genotype frequencies of the three polymorphic sites *14-bp ins-del*, *+3142C/G*, and *+3187A/G* were estimated by direct counting using the GENEPOP software version 3.4 [34]. The two-tailed Fisher exact test was used to compare the allele and genotype frequencies between HIV^+^ and healthy women, and between HIV^+^ women co-infected with HPV or not. The association between diplotype frequencies and aneuploidy were estimated by logistic regression. Results were considered significant at *p* < 0.05. Bonferroni correction was performed when necessary. Contingency tables were analyzed using the software GraphPad Prism version 5.01 for Windows (GraphPad Software, San Diego, CA, www.graphpad.com).

## Results

### Clinical features of the studied patients

In the present study, we evaluated 226 women from a HIV-infected cohort that had been previously studied by our research group and had shown low frequency of high-grade cervical lesion and high adherence to antiretroviral therapy [27], which is provided at no cost to the patient by the Brazilian government. Unfortunately, the research group did not have access to the histological reports of all women that participated in the study because the program for cervical cancer screening subsidized by the Brazilian Ministry of Health determines that histological evaluation must only be performed in women who had cytological abnormalities and atypical colposcopy findings what was not the case for several of the studied patients. Details of demographic and clinical features of HIV^+^ women co-infected or not with HPV are shown in Table 1. HIV^+^/HPV^+^ women presented a 5.5-fold higher risk of altered cervical cytology (*p* < 0.000) and a 2.0-fold higher risk of chromosomal aberration in cervical cells (*p* = 0.036) than HIV^+^/HPV^-^ women.

**Table 1:** Clinical and epidemiological characteristics of HIV-infected women according the HPV co-infection status attending three reference centers for HIV/AIDS in Recife, northeast of Brazil.

	HIV^+^						
Characteristics	HPV^+^		HPV^-^		*p*	OR	95% Cl
	N = 82	(%)	N = 144	(%)			
**Age (median = 35 years)**							
≥ 34 years	36	46.2	78	58.6	0.087	0.60	0.34–1.06
<34 years	42	53.8	55	41.4			
Total	78	100.0	133	100.0			
**Presence of histological alterations**							
Yes	9	60.0	3	37.5	0.4003	2.50	0.42–14.61
No	6	40.0	5	62.5			
Total	15	100.0	8	100.0			
**Presence of cytological alterations**							
Yes	20	29.9	9	7.1	**< 0.000**	**5.53**	**2.35–13.03**
No	47	70.1	117	92.9			
Total	67	100.0	126	100.0			
**CD4**^**+**^ **T lymphocyte count**							
<200/mm^3^	10	16.4	7	6.5	0.0607	2.80	1.00–7.79
≥200/mm^3^	51	83.6	100	93.5			
Total	61	100.0	107	100.0			
**Use of anti-retrovirus treatment**							
No	16	25.4	19	17.0	0.237	1.67	0.78–3.53
Yes	47	74.6	93	83.0			
Total	63	100.0	112	100.0			
**Cellular ploidy**							
Aneuploidy	22	31.4	24	18.2	**0.036**	**2.06**	**1.05–4.03**
Diploidy	48	68.6	108	81.8			
Total	70	100.0	132	100.0			

HIV^+^, women infected by Human Immunodeficiency Virus; HPV^+^, women infected by Human Papilloma Virus; (-) Not infected women; N, number of individuals; OR, odds ratio; CI, confidence interval. *P* was estimated by Fisher's exact two-tailed test. Cytological alterations: atypical squamous cells of undetermined significance (ASCUS), atypical squamous cells-cannot exclude high-grade squamous intraepithelial lesion (ASC-H), low-grade squamous intraepithelial lesion (LGSIL), and high-grade squamous intraepithelial lesion (HGSIL). Histological alterations: cervical intraepithelial neoplasia (CIN) I, CIN II, and CIN III.

Note: In most of HIV-infected patients, cervical biopsies were not performed, because the program for cervical cancer screening subsidized by the Brazilian Ministry of Health determines that histological evaluation must only be executed in women who had cytological abnormalities and atypical colposcopy findings.

### *HLA-G* 3’ UTR polymorphisms

First, we compared the frequency of the *HLA-G* 3’UTR variants between all the HIV^+^ women (n = 226) and the healthy women (n = 138). The *+3142G* allele (*p* = 0.049) and the *+3142GG* genotype (*p* = 0.047) were overrepresented in the HIV^+^ women, while the *+3187G* allele (*p* = 0.028) and the *+3187GG* genotype (*p* = 0.026) predominated among healthy women. No significant differences were observed for the *14-*bp *ins/del* polymorphism between HIV^+^ and healthy women (Table 2).

**Table 2:** Allelict and genotypic frequencies of HLA-G 3’ untranslated region (3’UTR) polymorphic sites of the *HLA-G* gene in the controls group and in HIV-infected women stratified according to the presence of HIV/HPV co-infection.

Polymorphisms sites	HIV^+^				HIV^-^		^a^HIV^+^		*vs*. Control	^b^HIV^+^		*vs*. Control	^c^HIV^+^		*vs*. Control	^d^HIV^+^ HPV^-^ *vs*. HPV^+^		
	HPV^+^		HPV^-^		Control		HPV^+^ and HPV^-^			HPV^+^			HPV^-^					
	N	(%)	N	(%)	N	(%)	*p*	OR	95% CI	*p*	OR	95% CI	*p*	OR	95% CI	*p*	OR	95% CI
**Alleles 14 bp *ins/del***	164		288		276													
*ins*	68	41.5	116	40.0	109	39.5	0.756	1.05	0.77–1.43	0.689	1.09	0.73–1.61	0.864	1.03	0.74–1.45	0.842	0.95	0.65–1.41
*del*	96	58.5	172	60.0	167	60.5	0.756	0.95	0.70–1.30	0.689	0.92	0.62–1.36	0.864	0.97	0.69–1.36	0.842	1.05	0.71–1.55
**Genotypes**	82		144		138													
*ins/ins*	15	18.3	25	17.3	21	15.2	0.566	1.20	0.67–2.13	0.575	1.25	0.60–2.58	0.633	1.17	0.62–2.20	0.858	0.94	0.46–1.90
*ins/del*	38	46.3	66	45.8	67	48.6	0.666	0.90	0.59–1.38	0.781	0.92	0.53–1.58	0.721	0.90	0.56–1.43	1.000	0.98	0.59–1.69
*del/del*	29	35.4	53	36.8	50	36.2	1.000	1.00	0.64–1.56	1.000	0.96	0.54–1.70	1.000	1.03	0.63–1.66	0.886	1.06	0.60–1.87
**Alleles *+3142C/G***	162		276		276													
*C*	64	39.5	92	33.9	119	43.1	**0.049**	**0.73**	**0.54–0.99**	0.484	0.86	0.58–1.28	**0.023**	**0.66**	**0.47–0.93**	0.215	0.77	0.51–1.14
*G*	98	60.5	184	66.1	157	56.9	**0.049**	**1.37**	**1.00–1.86**	0.483	1.16	0.78–1.72	**0.023**	**1.52**	**1.07–2.14**	0.215	1.31	0.87–1.95
**Genotypes**	81		138		138													
*CC*	17	21.0	20	15.0	29	21.0	0.331	0.76	0.44–1.31	1.000	0.99	0.51–1.96	0.207	0.64	0.34–1.19	0.263	0.64	0.31–1.30
*CG*	30	37.0	52	37.9	61	44.2	0.223	0.76	0.49–1.16	0.322	0.74	0.42–1.30	0.327	0.76	0.47–1.23	1.000	1.03	0.58–1.81
*GG*	34	42.0	66	47.1	48	34.8	**0.047**	**1.58**	**1.01–2.45**	0.313	1.35	0.77–2.38	**0.038**	**1.72**	**1.06–2.79**	0.483	1.27	0.73–2.20
**Alleles *+3187A/G***	158		276		264													
*A*	111	70.3	219	79.5	180	68.2	**0.028**	**1.48**	**1.05–2.09**	0.665	1.10	0.72–1.7	0.003*	**1.79**	**1.21–2.65**	**0.036**	**1.63**	**1.04–2.55**
*G*	47	29.7	57	20.5	84	31.8	**0.028**	**0.68**	**0.48–0.95**	0.665	0.91	0.59–1.40	0.003*	**0.56**	**0.38–0.82**	**0.036**	**0.61**	**0.39–0.96**
**Genotypes**	79		138		132													
*AA*	40	50.6	88	63.8	67	50.8	0.149	1.40	0.90–2.16	1.000	0.99	0.57–1.74	**0.036**	**1.71**	**1.05–2.80**	0.064	1.72	0.98–3.01
*AG*	31	39.2	43	31.2	46	34.8	0.908	0.97	0.61–1.52	0.556	1.21	0.68–2.15	0.605	0.85	0.51–1.41	0.237	0.70	0.39–1.25
*GG*	8	10.1	7	05.0	19	14.4	**0.026**	**0.44**	**0.22–0.90**	0.403	0.67	0.28–1.21	**0.012**	**0.32**	**0.13–0.78**	0.269	0.51	0.18–1.45

HIV^+^, women infected by Human Immunodeficiency Virus; HPV^+^, women infected by Human Papilloma Virus; (-) Not infected women; N, number of individuals; OR, odds ratio; CI, confidence interval. Statistics used was Fisher's exact two-tailed test. Bold values denote differences statistically significant with *P* < 0.05. (*) P-value statistically significant after Bonferroni correction.

^a^ Comparison of genetic frequencies between overall HIV-infected women and immunocompetent women (control group)

^b^ Comparison of genetic frequencies between HIV-infected women with HIV/HPV co-infection and control group

^c^ Comparison of genetic frequencies between HIV-infected women without HIV/HPV co-infection and control group

^**d**^ Comparison of genetic frequencies between HIV-infected women without and with HIV/HPV co-infection

Secondly, we compared the frequency of the same *HLA-G* 3’UTR variants between HIV^+^/HPV^+^ women (n = 82) and healthy controls (n = 138), and no significant results were observed. In contrast, when HIV^+^/HPV^-^ women (n = 144) were compared to healthy controls, several differences were observed: the *+3142G* allele (*p* = 0.023) and the *+3142GG* genotype (*p* = 0.038) were associated with susceptibility to HIV infection, and the *+3187A* alleles (*p* = 0.003) and *+3187AA* genotype (*p* = 0.036) were overrepresented in HIV^+^/HPV^-^ patients. The power of association between the *+3142C/G* and *+3187A/G* polymorphisms and susceptibility to HIV infection increased when the HIV^+^/HPV^+^ women were excluded from the HIV^+^ group. The *+3187G* allele was overrepresented in HIV^+^/HPV^+^ women (*p* = 0.036). The *+3187A/G* allelic frequencies of HIV^+^/HPV^-^ women and healthy women were significantly different (*p* = 0.018) even after correction for multiple comparison using the Bonferroni test, considering three polymorphic sites and two alleles. No significant differences were observed regarding the *14-bp ins/del* polymorphism between HIV^+^/HPV^-^ and control women. The *HLA-G +3142GG/+3187AA* diplotype showed a trend increase (*p* = 0.071) in the group of HIV^+^ women compared to healthy women (Table 3).

**Table 3:** Diplotypes frequencies of HLA-G 3’ untranslated region (3’UTR) polymorphic sites of the *HLA-G* gene in the controls group and in HIV-infected women.

*HLA-G* *+3142/+3187* diplotypes	^a^HIV^+^		HIV^-^		^a^HIV^+^		*vs*. Control
	HPV^+^ and HPV^-^		Control		HPV^+^ and HPV^-^		
	N	(%)	N	(%)	*p*	OR	95% CI
*+3142CC/+3187AA*	8	03.0	2	01.5	0.330	2.50	0.52–11.96
*+3142CG/+3187AA*	25	11.0	21	15.9	0.257	0.69	0.37–1.29
*+3142GG/+3187AA*	94	44.3	44	33.3	0.071	1.54	0.98–2.42
*+3142CC/+3187GG*	14	06.4	16	12.1	0.078	0.50	0.24–1.07
*+3142CG/+3187GG*	0	00.0	3	02.2	NA	NA	NA
*+3142GG/+3187GG*	0	00.0	0	00.0	NA	NA	NA
*+3142CC/+3187AG*	15	06.9	9	06.8	1.000	1.02	0.43–2.40
*+3142CG/+3187AG*	57	27.1	37	28.2	0.804	0.92	0.56–1.50
*+3142GG/+3187AG*	3	01.3	0	00.0	NA	NA	NA

HIV^+^, women infected by Human Immunodeficiency Virus; HPV^+^, women infected by Human Papilloma Virus; (-) Not infected women; N, number of individuals; OR, odds ratio; CI, confidence interval; NA, stands for not applicable.

^a^ Comparison of genetic frequencies among overall HIV-infected women independent of the presence of HIV/HPV co-infection and immunocompetent women (control group)

Statistics used was Fisher's exact two-tailed test to compare each group with the others combined.

### Association of *HLA-G* 3′ UTR polymorphisms with aneuploidy in cervical cell

We recently reported a lack of association between aneuploidy in cervical cells and HIV^+^/HPV^+^ co-infection (*p* = 0.22), low-risk (*p* = 0.43) or high-risk HPV (*p* = 0.17), and cytological alteration (*p* = 0.35) in the HIV-infected women cohort [28]. However, aneuploidy was associated with histological lesion (*p* = 0.03) [28]. Considering that aneuploidy is a phenomenon associated with cervical cell transformation, and HLA-G is an immune checkpoint protein with inhibitory effect on several immune cells, we hypothesized that women harboring *HLA-G* 3’UTR alleles associated with high HLA-G expression may also have higher risk to develop aneuploidy.

Thirty four samples were excluded from HIV^+^ group (n = 226) because they either had no data on cellular ploidy (n = 24) or lacked polymorphism information (n = 10). Comparing the diplotype distribution of women with cervical cells presenting or not aneuploidy, we observed that the +*3142GG/+3187AA* diplotype was the most prevalent in both aneuploid (45.45%) and diploid (43.92%) cervical cells. Considering the double heterozygous +*3142CG*/+*3187AG* diplotype as reference and using the logistic regression model, we observed that: (i) diplotypes defined by +*3142CC/+3187G****X***, where X is either the *G* or *A* allele, showed no differences related to ploidy (Table 4), (ii) the double heterozygous diplotype (+*3142CG/+3187AG*) was the second most prevalent in the diploid group (29.05%) and was underrepresented in the aneuploidy group (15.91%), and (iii) the frequency of the +*3142C****X****/+3187AA* diplotype was significantly higher than the frequency of the double heterozygous haplotypes in the aneuploid group, and this association was strengthened when HPV infection was considered in the model. Unfortunately, we were not able to investigate the influence of HPV-risk classification on the relationship between +*3142C****X****/+3187AA* diplotype and aneuploidy using the logistic regression model due to a small sample size after data stratification by HPV risk.

**Table 4:** Linear regression between the *HLA-G +3142/+3187* diplotypes and presence of aneuploidy in cervical cells from HIV-infected women.

*HLA-G* *+3142/+3187* diplotypes	Aneuploidy				OR	Overall risk			OR	Adjusted risk for HPV		
	Yes		No			95% CI		*p*		95%CI		*p*
	N	(%)	N	(%)		2.5%	97.5%			2.5%	97.5%	
*CG+AG*	7	15.91	43	29.05	1.00				1.00			
*CC+GG*	3	6.82	10	6.76	1.84	0.35	8.01	0.4298	1.63	0.31	7.24	0.5307
*GG+AA*	20	45.45	65	43.92	1.89	0.76	5.17	0.1858	2.01	0.81	5.57	0.1506
*CC+AA*	3	6.82	3	2.03	**6.14**	0.97	39.74	**0.0467**	7.40*	1.14	49.36	**0.0310**
*CC+AG*	3	6.82	11	7.43	1.68	0.32	7.18	0.5018	1.52	0.29	6.65	0.5873
*CG+AA*	8	18.18	16	10.81	**3.07**	0.96	10.15	**0.0592**	3.21**	0.99	10.80	**0.0526**
Adjusted risk for HPV									2.06	1.00	4.24	**0.0486**

N, number of individuals; *p*, significance; OR, odds ratio; CI, confidence interval. The presence of the HPV increases 20.5% (*) and 4.6% (**) the risk of aneuploidy in women carrying the +*3142C* allele in homozygosis and heterozygosis, respectively.

We also evaluated whether oncotic cytology was associated with *HLA-G* polymorphisms in HIV^+^ women (Table 5). No significant differences were observed between the +*3142/+3187* diplotypes and the presence of cytological alterations (*p* = 0.407), even after data stratification by presence (*p* = 0.324) or absence (*p* = 0.639) of HPV infection. No association (*p* = 0.603) was observed between the +*3142/+3187* diplotypes and CD4^+^ T lymphocyte count (Table 6).

**Table 5:** *HLA-G +3142/+3187* diplotypes, cytological abnormalities and stratified case analysis according to the risk of HPV infection in HIV-infected women.

*HLA-G +3142/+3187* diplotypes	Total				Stratification by risk to HPV								*p*–value^2^
	Cytological alterations				HPV^+^				HPV^-^				
					Cytological alterations				Cytological alterations				
	Positive		Negative		Positive		Negative		Positive		Negative		
	N	(%)	N	(%)	N	(%)	N	(%)	N	(%)	N	(%)	
*CG+AG*	0	0.00	8	5.19	0	0.00	2	4.55	0	0.00	6	5.45	0.1988
*CC+GG*	3	10.71	9	5.84	3	15.79	2	4.55	0	0.00	7	6.36	
*CC+AA*	1	3.57	12	7.79	1	5.26	6	13.64	0	0.00	6	5.45	
*CC+AG*	4	14.29	19	12.34	3	15.79	3	6.82	1	11.11	16	14.55	
*CG+AA*	5	17.86	46	29.87	4	21.05	16	36.36	1	11.11	30	27.27	
*GG+AA*	15	53.57	60	38.96	8	42.11	15	34.09	7	77.78	45	40.91	
***p*—value**^**1**^	0.4069				0.3238				0.6386				

Positive, presence of cytological alterations; Negative, absence of cytological alterations; HPV+, women infected by Human Papilloma Virus; (-) Not infected women; N, number of individuals; OR, odds ratio; CI, confidence interval.

*p*—value^1^ = Chi-square Test / Exact Fisher Test.

*p*—value^2^ = Mantel-Haenszel.

**Table 6:** Frequency distribution of the *HLA-G +3142/+3187* diplotypes in relation to the CD4^+^ T lymphocyte count values in HIV-infected women.

*HLA-G* *+3142/+3187* diplotypes	CD4^+^ T lymphocyte count								
	N	(%)	Minimum	Maximum	Mean	Median	Standard deviation	Standard error	*p*
*CG+AG*	45	25.71	9	1.379	481.96	450.00	340.53	50.76	0.6030
*CC+GG*	14	8.00	9	745.00	407.43	377.50	205.73	54.98	
*CC+AA*	5	2.86	5	1.622	652.80	548.00	591.26	264.42	
*CC+AG*	11	6.29	9	956.00	463.82	467.00	298.83	90.10	
*CG+AA*	22	12.57	9	1.090	551.09	543.50	270.79	57.73	
*GG+AA*	78	44.57	9	1.339	460.13	450.50	313.85	35.54	

N, number of individuals.

## Discussion

The role of HLA-G in HIV infection is controversial due to several reasons, including disease diversity, presence of underlying disorders, patient ethnicity and treatment regimens. Although there are eight well-known polymorphic sites in the *HLA-G* 3’UTR region, in our study, the frequencies of only the +*3142C/G* and +*3187A/G* sites were statistically different between healthy controls and HIV^+^ patients. We believe that by including a screening step for selecting only *HLA-G* polymorphic sites that contribute to susceptibility to viral infection, we minimize the inclusion of genetic factors not related to it. These two polymorphic sites are target for microRNA, important post-transcriptional gene expression regulators, which expression profiles have been demonstrated to diverge according to cell type and stimulus [35]. Nevertheless, we also discussed the 14-bp *ins/del* allele and genotype frequencies, because of its influence on HLA-G levels in HIV^+^ patients, as has been demonstrated in previous studies [6, 8, 17].

We also understand that environmental pressures may affect the evolution of the immune system differentially and therefore the polymorphic sites we studied may not be representative for other populations from different locations. In a previous study, we evaluated *HLA-G* 3’UTR polymorphic sites allele, genotype and haplotype frequencies in healthy blood donors from a northeastern and southeastern Brazilian regions, and we found that the distribution of the *HLA-G* 3'UTR 14bp-*ins/del*, +*3142C/G*, and +*3187A/G* were similar [36]. However, comparing these published data with the distribution frequency of these polymorphic sites observed in healthy women group reported in the present study, no differences were found for the alleles and genotypes regarding to the northeastern population; in contrast, we found a higher frequency of +*3187GG* genotype (14.4% vs. 4.5%, respectively) in the healthy women group of the present study than in healthy blood donors from southeast Brazil [32]. This findings could be related to the contribution of different ethnic groups to the constitution of the Brazilian populations [36]. It is noteworthy that a study on the Brazilian ancestry based on mitochondrial DNA showed a major contribution of African ancestry in northeast Brazil [37].

There are few reports in the literature regarding the association between presence of *HLA-G* 3’UTR polymorphisms to susceptibility to HIV and HPV infection, and the 14-bp *ins/del* site is by far the most studied [8, 17, 18]. The frequency of +*3187A* allele in homozygosis was found in only one study on HIV vertical transmission, and it was not appropriated for comparison [38]. Future studies may confirm whether the presence of the +*3142G* and +*3187A* alleles are associated with risk of HIV infection in different populations.

Nevertheless, HIV infection increases the production of HLA-G by naïve T CD8^+^ cells and increases effector and memory cell lines [39]. The virus itself induces high levels of sHLA-G [40] and the antiretroviral therapy, during the immunological reconstitution, down-regulates HLA-G [41]. The *HLA-G 14-bp ins* allele, which is associated with low levels of sHLA-G in healthy Caucasian individuals [7], has been associated with low levels of sHLA-G in African HIV-infected individuals [6]. In contrast, the presence of the *14-bp del/del* genotype, which has been associated with high sHLA-G levels in healthy Caucasians [7], was associated with high expression of *HLA-G* mRNA, high viral load, lower CD4^+^ T lymphocytes counts and low survival rate in HIV-infected Zimbabwean women [8]. The *HLA-G* 3’UTR *14-bp ins* allele, the *ins/ins* genotype, the *14-bp insertion/+3142G (insG)* haplotype and the *insG/insG* diplotype were overrepresented in African-derived patients HIV^+^ from Southern Brazil, irrespective of the underlying disorders; but, the same was not observed in European-derived Brazilians [17]. In the present study, the associations with the *14-bp* polymorphism was not observed in the HIV^+^ women co-infected or not with HPV from the state of Pernambuco, northeast Brazil, which have a higher African-ancestry than Southern Brazilians.

The role of HLA-G on HPV mono-infection has not been completely elucidated yet. Gimenes et al. (2014) [42] recently reviewed studies correlating the association between HLA-G polymorphisms and cervical carcinogenesis. The presence of high levels of *HLA-G* mRNA in cervical lesion was reported to contribute to their progression in HPV 16/18 infected patients [21,43,44]. The *HLA-G* 3’UTR diversity has only been evaluated in patients with HPV mono-infection, including the *14-bpdel*/+*3142C* haplotype in Italian women [9], and the *+3142C* allele in Taiwanese women [45], which were associated with increased risk for developing HPV cervical cancer. For the HIV/HPV co-infection, we observed that the +*3142G* and *+3187A* alleles were associated with predisposition to HIV infection independent of the presence of HIV/HPV co-infection. In addition, the double doses, +*3142GG* and +*3187AA*, also exhibited a trend increased risk for HIV infection, independent of the HIV/HPV co-infection. Considering that the *+3142G* allele reduces *HLA-G* mRNA expression by the action of specific microRNAs [10], and that the *+3187A* allele decreases the *HLA-G* mRNA stability due to the proximity to an AU motif [12], our results indicate that susceptibility to HIV, irrespective of HPV infection, may be favored by low HLA-G expression.

Since HIV and HPV present distinct biological behavior, we further analyzed the relationship between the *HLA-G* 3’UTR polymorphic sites and aneuploidy, which may represent the initial step for progression towards cervical malignancy [46]. In the present study, the *+3142C****X***/+*3187AA* diplotype was associated with aneuploidy of cervical cells; i.e., the presence of +*3142CC* and +*3142CG* increased by 7.40-fold and 3.21-fold the risk for cervical lesion, respectively. Noteworthy, the association with +*3142C/G* polymorphic site was only observed when the +*3187AA* genotype was present as well.

Although the *+3142C* allele was underrepresented in the HIV^+^ patients compared to healthy women, this allele was associated with aneuploidy in cervical cell in the HIV^+^ women. Studies have shown that the *+3142C* allele and *+3142CC* genotype increase sHLA-G production, which has been associated with HPV infection and risk to develop cervical cancer [9,45]. In this context, we showed that the presence of HPV infection increased the contribution of the +*3142C* allele to the susceptibility of the +*3142CX*/+*3187AA* diplotype to aneuploidy by 20.5% (in homozygosis) and 4.6% (in heterozygosis) (Table 4); however, it was not associated with cytological abnormalities. We have previously shown the relationship between the presence of cervical tissue lesions and the DNA index, but cytological alteration was not predictive of progression of cervical lesion [28].

In resume, we showed that the +*3142G* and +*3187A* alleles, which are related to low HLA-G expression [10,12], were associated with predisposition to HIV infection independent of the presence of HIV/HPV co-infection, but HIV induces the production of HLA-G in untreated patients through unclear mechanisms [41]. Perhaps, the increased HLA-G level dependent of HIV infection is one of the mechanisms behind the association between HIV infection and high-risk of HPV co-infection [47,48], and increased risk of cytological abnormalities and cervical cancer [49,50]. Indeed, we showed that in HIV^+^ women the +*3142C* allele and the +*3142CC* genotype, which are related to increased sHLA-G production, were associated to the presence of aneuploidy in cervical cells in an allele dose-dependent effect. Our results corroborate previous studies that showed that the +*3142C* allele is associated to HPV infection and to a higher risk to develop cervical cancer in HIV^-^ women [9,45]. Taken together, we conclude that aneuploidy, one of the first steps towards the development of cervical malignancies [46], is favored by *HLA-G* alleles related to high HLA-G production, because the inhibitory effect of the HLA-G on immune cells modulates the immune cellular response, which then contribute to replication and persistence of HPV infections, and increases the chance of cervical lesion progression.

Our study dealt with limitations: we did not quantify the HLA-G levels, as well as, the fact that histological reports were not available for all women that participated in the study because the Brazilian Ministry of Health’s protocol for screening cervical cancer, defines that histological evaluation must only be performed in women who had cytological abnormalities and atypical colposcopy findings. In addition, unfortunately, some women did not return to the ambulatory clinic for the histological exams or reassessment, and, consequently, their exam results could not be provided to the research group, including seven patients with altered cytology for whom the histology results were not recorded.
